# Electroacupuncture Exerts Analgesic Effects by Restoring Hyperactivity via Cannabinoid Type 1 Receptors in the Anterior Cingulate Cortex in Chronic Inflammatory Pain

**DOI:** 10.1007/s12035-023-03760-7

**Published:** 2023-11-13

**Authors:** Junshang Wu, Libo Hua, Wenhao Liu, Xiaoyun Yang, Xiaorong Tang, Si Yuan, Sheng Zhou, Qiuping Ye, Shuai Cui, Zhennan Wu, Lanfeng Lai, Chunzhi Tang, Lin Wang, Wei Yi, Lulu Yao, Nenggui Xu

**Affiliations:** 1 South China Research Center for Acupuncture and Moxibustion, Medical College of Acu-Moxi and Rehabilitation, Guangzhou University of Chinese Medicine, Guangzhou, China; 2 Department of Acupuncture and Moxibustion, the Second Affiliated Hospital of Guangzhou University of Chinese Medicine, Guangzhou, China; 3 Research Institute of Acupuncture and Moxibustion, Shandong University of Traditional Chinese Medicine, Jinan, China; 4 Department of Rehabilitation MedicineThe Third Affiliated Hospital, Sun Yat-Sen University, Guangzhou, China; 5 Acupuncture and Meridian Research Institute, Anhui Academy of Chinese Medicine, Anhui, China

**Keywords:** Inflammatory pain, Electroacupuncture, Anterior cingulate cortex, Cannabinoid type 1 receptor, Analgesia

## Abstract

As one of the commonly used therapies for pain-related diseases in clinical practice, electroacupuncture (EA) has been proven to be effective. In chronic pain, neurons in the anterior cingulate cortex (ACC) have been reported to be hyperactive, while the mechanism by which cannabinoid type 1 receptors (CB1Rs) in the ACC are involved in EA-mediated analgesic mechanisms remains to be elucidated. In this study, we investigated the potential central mechanism of EA analgesia. A combination of techniques was used to detect the expression and function of CB1R, including quantitative real-time PCR (q-PCR), western blot (WB), immunofluorescence (IF), enzyme-linked immunosorbent assay (ELISA), and in vivo multichannel optical fibre recording, and neuronal activity was examined by in vivo two-photon imaging and in vivo electrophysiological recording. We found that the hyperactivity of pyramidal neurons in the ACC during chronic inflammatory pain is associated with impairment of the endocannabinoid system. EA at the Zusanli acupoint (ST36) can reduce the hyperactivity of pyramidal neurons and exert analgesic effects by increasing the endocannabinoid ligands anandamide (AEA), 2-arachidonoylglycerol (2-AG) and CB1R. More importantly, CB1R in the ACC is one of the necessary conditions for the EA-mediated analgesia effect, which may be related to the negative regulation of the N-methyl-D-aspartate receptor (NMDAR) by the activation of CB1R downregulating NR1 subunits of NMDAR (NR1) via histidine triad nucleotide-binding protein 1 (HINT1). Our study suggested that the endocannabinoid system in the ACC plays an important role in acupuncture analgesia and provides evidence for a central mechanism of EA-mediated analgesia.

**Supplementary Information:**

The online version contains supplementary material available at 10.1007/s12035-023-03760-7.

## Background

The International Association for the Study of pain (IASP) defines chronic pain is an unpleasant sensory and emotional experience associated with, or resembling that which is associated with, actual or potential tissue damage [1]. More than 30% of the global population suffers from chronic pain, which severely lowers patients' quality of life, causes disability, and imposes a heavy economic burden on individuals and society [2, 3]. However, current clinical pharmacological treatments for chronic pain have adverse effects, such as drug resistance and addiction [4, 5]. As a traditional therapy, electroacupuncture (EA) has been proven to be effective in analgesia and has been widely used in clinical practice [6, 7]. The mechanism underlying EA-mediated analgesia has attracted increasing interest from researchers. Previous evidence suggested that EA could exert analgesic effects through multiple bioactive chemicals, such as opioids, adenosine, serotonin, and norepinephrine [8, 9]. Meanwhile, most of studies have focused on periphery, spinal, and subcortical regions including the periaqueductal grey (PAG), rostral ventromedial medulla (RVM) and thalamus [10, 11]. However, the role of cortical areas in EA-mediated analgesia is not fully understood [12]. Zusanli acupoint (ST36) is a typical acupoint that is widely used to treat for pain-related diseases in clinical practice and preclinical experiments [8, 13, 14]. EA at ST36 was evidenced to promote opioid peptide, serotonin, and dopamine, as well as exert anti-inflammatory analgesic effects through the cholinergic pathway and vago-adrenal axis pathway [15, 16]. Therefore, ST36 was chosen as the treatment for the pain model in the study. The anterior cingulate cortex (ACC) is a critical brain region for the perception of pain and affect, and neurons in the ACC can be activated by various forms of pain stimuli, such as mechanical and thermal pain [17, 18]. Current studies have shown that the hyperexcitability of pyramidal neurons in the ACC disrupts the excitation-inhibition balance in chronic pain states and the inhibition of this hyperexcitability is sufficient to produce analgesia [19–21]. Optogenetic inhibition of pyramidal neurons could have an analgesic effect in the complete Freund’s adjuvant (CFA)-induced chronic pain mouse model [22]. Therefore, we sought to explore whether this disrupted neuronal activity could be modulated during EA treatment for chronic pain. The cannabinoid type 1 receptor (CB1R) is one of the most expressed G-protein-coupled receptor subtypes in the central nervous system (CNS) [23, 24], with an enrichment in the ACC [25, 26]. Numerous studies have shown that CB1R is involved in EA-mediated analgesic processes in different brain regions such as the striatum, PAG, and primary somatosensory cortex (S1) [27, 28]. The pharmacologic experiments using CB1R agonists, such as N-palmitoylethanolamide (PEA) and cannabidiol, suggested that CB1R in the ACC could contribute to the analgesic effects [29, 30]. The mechanism by which CB1R in the ACC participates in EA-mediated analgesia remains to be elucidated.

In this study, the results indicated that EA-mediated analgesia in the chronic inflammatory pain was associated with an impaired endogenous cannabinoid system in the ACC and that CB1R was involved in the regulation of the hyperactivation of pyramidal neurons. Finally, our results suggested that EA could regulate the hyperactivity of pyramidal neurons in the ACC by CB1R downregulating NR1 subunits of N-methyl-D-aspartate receptor (NMDAR) via histidine triad nucleotide-binding protein 1 (HINT1).

## Results

### EA Exerted Analgesic Effects in the CFA-induced Pain Model and Restored the Neuronal Hyperactivity of the ACC

**Fig. 1:**
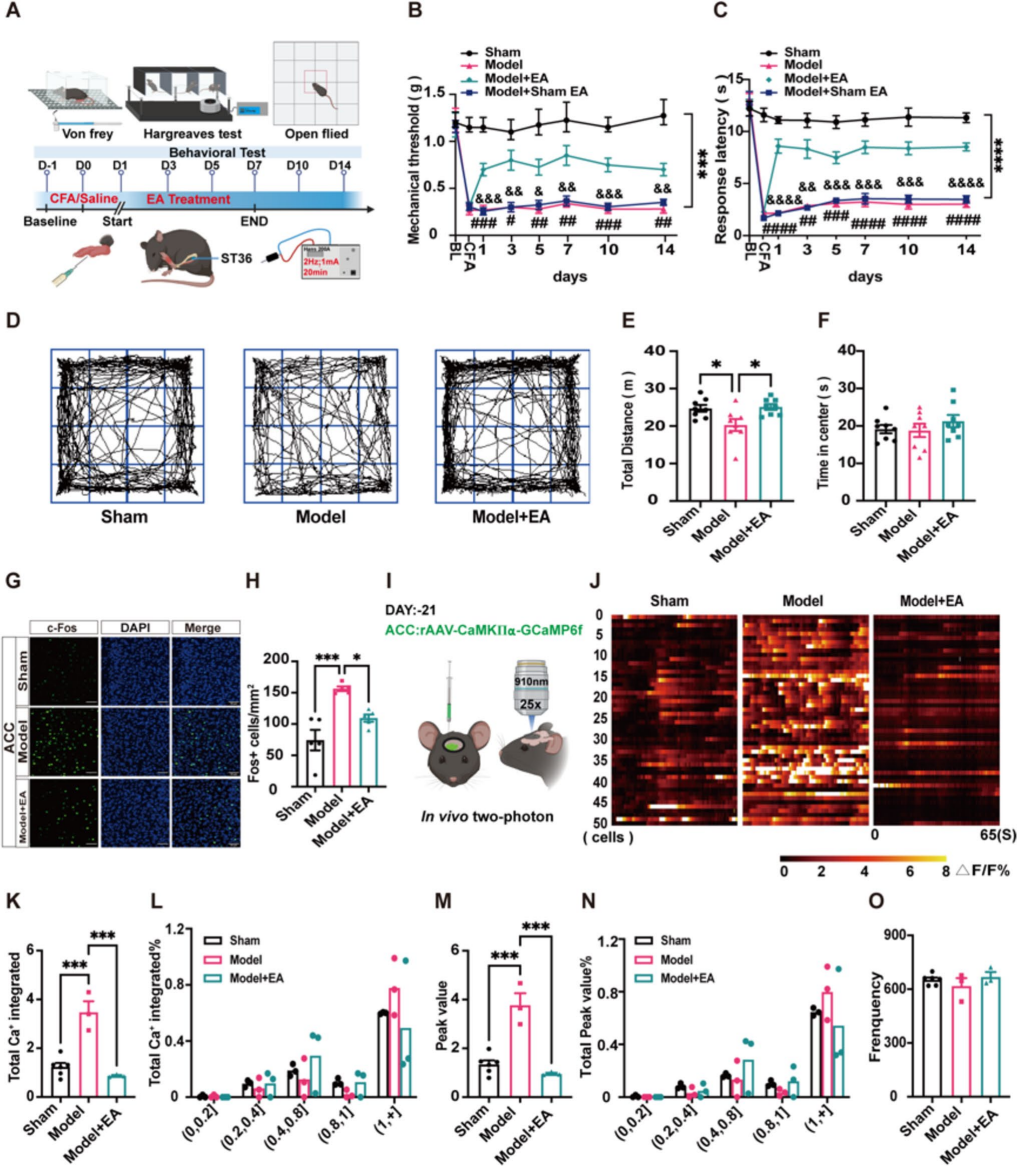
EA may exert analgesic effects by decrease of abnormal neuronal activity in ACC**.** (**A**) A timeline of EA treatment and behavioral testing. Male C57 mice were injected with CFA on day 0, treated with EA (2 Hz, 1 mA, 20 min) from day 1 to day 7, and tested with von Frey mechanical threshold and thermal pain latency on days -1, 0, 1, 3, 5, 7, 10, and 14. (**B**) Mechanical threshold was detected using von Frey filaments. EA significantly reversed the decrease in mechanical thresholds in model mice, whereas Model + sham EA had no therapeutic effect. The baseline was no statistical difference between the four groups, Sham group: 1.2 ± 0.214, Model group: 1.22 ± 0.36, Model + EA group: 1.18 ± 0.36, Model + Sham EA group: 1.16 ± 0.207. (Two-way ANOVA with Bonferroni test, F _(21, 196)_ = 5.46, *P* < *0.0001.* *N =* 8 mice for each group. *** Sham group vs. Model group and Model + Sham EA, respectively; #, ##, ### Model + EA group vs. Model group; &, &&, &&& Model + EA group vs. Model + Sham EA group). (**C**) The paw withdrawal latency was measured by Hargreaves' test. EA significantly reversed the decrease in thermal pain latency in model mice, whereas sham EA had no therapeutic effect. The baseline was no statistical difference between the four groups, Sham group: 12.21 ± 2.0, Model group: 13.17 ± 1.54, Model + EA group: 12.65 ± 1.23, Model + Sham EA group: 12.65 ± 3.36. (Two-way ANOVA with Bonferroni test, F _(21, 196)_ = 14.76, *P* < *0.0001.* *N =* 8 mice for each group. **** Sham group vs. Model group and Model + Sham EA, respectively; ##, ###, #### Model + EA group vs. Model group; &&, &&&*,* &&&& Model + EA group vs. Model + Sham EA group). (**D**) The movement traces of mice in open field for 10 min. (**E**) Statistical graph showing the total travel distance of mice in open field. EA significantly reversed the reduction in the total travel distance of the model mice. Sham group: 24.72 ± 0.91, Model group: 20.27 ± 1.6, Model + EA group: 25.10 ± 0.77. (One-way ANOVA with Bonferroni test, F _(2, 21)_ = 5.4, *P* < *0.05.* *N =* 8 mice for each group. * vs. Sham group and Model + EA group). (**F**) Statistical graph showing the time of mice in the central of open field. No difference in residence time in the central region between the three groups of mice. Sham group: 19.14 ± 1.12, Model group: 18.79 ± 1.78, Model + EA group: 21.27 ± 1.64. (One-way ANOVA with Bonferroni test, F _(2, 21)_ = 0.76, *P* > *0.05.* *N =* 8 mice for each group.). (**G**) Confocal imaging of c-Fos expression in ACC, Scale bars: 50 μm. (**H**) The number of c-Fos^+^ neurons in ACC of three groups. EA reverses elevated c-Fos-positive neurons in ACC. Sham group: 74.3 ± 16.39, Model group: 156.27 ± 3.62, Model + EA group: 109.4 ± 5.86. (One-way ANOVA with Bonferroni test, F _(2,12)_ = 16.04, *P* < *0.001.* *N =* 5 mice for each group. *** vs. Sham group, * vs. Model + EA group). (**I**) Schematic diagram of in vivo two-photon imaging of CaMKIIα neurons expressing GCaMP6fs by ACC injection of rAAV-CaMKIIα-GCaMP6fs virus. (**J**) Heat maps showed changes in Ca^2+^ activity in 50 neurons at layer L2/3 of ACC in the three groups of mice within 65 s, respectively. The color code on the bottom indicates ΔF/F (%). (**K**) The mean total integrated Ca^2+^ activity of the three groups. EA alleviates the increase in neuronal mean total integrated Ca^2+^ activity in ACC of model mice. Sham group: 1.24 ± 0.16, Model group: 3.48 ± 0.45, Model + EA group: 0.87 ± 0.55. (One-way ANOVA with Bonferroni test, F _(2,9)_ = 29.08, *P* < *0.001*. *** vs. Sham group and Model + EA group, respectively). (**L**) Statistical plots of different frequency distributions of total integrated Ca^2+^ activity. (**M**) The mean peak Ca^2+^ activity in three groups of mice. EA alleviates the increase in neuronal mean peak Ca^2+^ activity in ACC of model mice. Sham group: 1.34 ± 0.16, Model group: 3.77 ± 0.85, Model + EA group: 0.95 ± 0.08. (One-way ANOVA with Bonferroni test, F _(2,9)_ = 29.9, *P* < *0.001*. *** vs. Sham group and Model + EA group, respectively). (**N**) The peak frequency distribution of Ca^2+^ activity. (**O**) Mean Ca^2+^ spike frequency of threes group. (I-O) *N =* 6, 3, 3 for Sham, Model, Model + EA group, respectively. Data are presented as mean ± SEM

The chronic inflammatory pain model was established by injecting complete Freund's adjuvant (CFA, 25 µl) into the plantar of the left hind limb of mice [31]. After 24 h of injection, EA treatment was manipulated on the left side of ST36 in mice for 7 consecutive days [32] (Fig. 1A). Mice with comparable baseline mechanical thresholds and thermal pain latencies were selected for further modelling. The results showed a significant decrease in the threshold, latency, and motility of the von Frey, thermal latency, and open field (Fig. 1B-F). EA at the ST36 acupoint significantly reversed malfunction, and the analgesic effect lasted for at least 7 days even after withdrawal of EA. In addition, EA increased the reduction in the total distance travelled by the mice after modelling with no alteration in the central time (Fig. 1B-F). The above results supported the analgesic effect of EA treatment on inflammatory pain.

To investigate the potential brain regions involved in EA-mediated analgesia, we first performed c-Fos staining on several potentially related brain regions, including the ACC, ventrolateral periaqueductal grey (vlPAG), medial prefrontal cortex (mPFC), lateral parabrachial nucleus (LPBN) and CA1 [33, 34]. EA reversed the increase in c-Fos positive neurons in the ACC of mice with chronic pain, and the decrease in the vlPAG. However, EA showed no obvious effect in the LPBN, mPFC and CA1 (Fig. 1G-H and Fig. S1 A-C). These results suggested that ACC was involved in chronic pain, and we further detected the neuronal activity in the ACC by in vivo two-photon Ca^2+^ imaging. We injected AAV-CaMKIIα-GCaMP6fs virus and performed modelling and EA treatment (Fig. 1I and Fig. S2 A-C). The results demonstrated that the Ca^2+^ signal in the ACC was significantly higher in the model mice than in the sham mice, and EA alleviated this hyperactivation (Fig. 1I-O). Overall, we speculated that EA could attenuate hyperactivity in the ACC to exert an analgesic effect.

### Hyperactivity in the ACC was Associated with Impaired Endocannabinoid Signalling

**Fig. 2:**
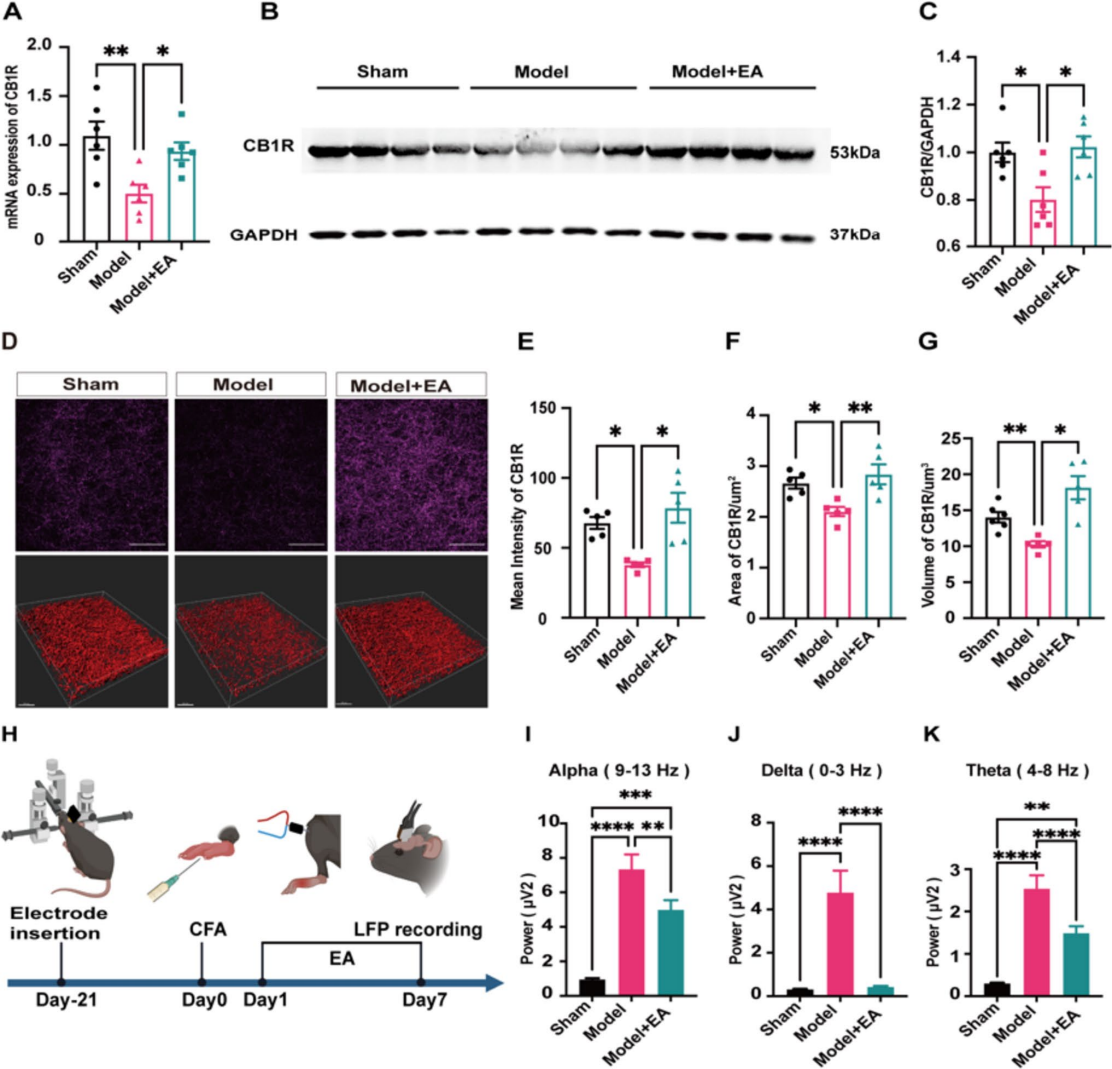
Endocannabinoids may be involved in electroacupuncture analgesia. (**A**) The mRNA level of CB1R in ACC was quantitatively analyzed. EA significantly increased CB1R mRNA downregulation in the ACC of model mice. Sham group: 1.09 ± 0.14, Model group: 0.5 ± 0.91, Model + EA group: 0.94 ± 0.90. (One-way ANOVA with Bonferroni test, F _(2,15)_ = 7.5, *P* < *0.01.* *N =* 6 mice for each group. ** vs. Sham group, * vs. Model + EA group). (**B**) Representative gel image of CB1R protein levels in ACC. (**C**) Statistical map of relative expression levels of CB1R protein. EA significantly increased CB1R protein downregulation in the ACC of model mice. Sham group: 1.0 ± 0.41, Model group: 0.8 ± 0.52, Model + EA group: 1.02 ± 0.44. EA significantly increased the fluorescence intensity of CB1R in the ACC of model mice. (One-way ANOVA with Bonferroni test, F _(2,15)_ = 7.06, *P < *0.05.* *N =* 6 mice for each group. * Sham group and Model + EA group, respectively). (**D**) The distribution of CB1R in ACC was displayed by immunofluorescence staining and 3D reconstruction using Imairs 9.0 software, Scale bars: 50 μm in immunofluorescence image and 30 μm in 3D reconstruction image. (**E**) Statistical graph of mean fluorescence intensity of CB1R in ACC. EA significantly increased the fluorescence intensity of CB1R in the ACC of model mice. Sham group: 67.82 ± 4.3, Model group: 37.91 ± 1.69, Model + EA group: 78.18 ± 10.92. (Dunnett T3 test, *N =* 5 mice for each group. * Sham group and Model + EA group, respectively). (**F**) The statistical plot represents the area of the CB1R in ACC. EA significantly increased the area of CB1R in the ACC of model mice. Sham group: 266621.32 ± 10803.03, Model group: 210534.81 ± 9188.47, Model + EA group: 283744 ± 19673.11. (One-way ANOVA with Bonferroni test, F _(2,12)_ = 7.51, *P* < *0.01.* *N =* 5 mice for each group. * vs. Sham group, ** vs. Model + EA group). (**G**) The statistical graph represents the volume of the CB1R in ACC. EA significantly increased the volume of CB1R in the ACC of model mice. Sham group: 145177.97 ± 6740.74, Model group: 102766.02 ± 4407.62, Model + EA group: 181473.25 ± 16117.87. (Tamhane T2 test, *N =* 5 mice for each group. ** vs. Sham group, * vs. Model + EA group). (**H**) A timeline of in vivo electrophysiological LFP recording. The electrodes were implanted in the ACC of mice by stereotaxic, and CFA was injected 21 days after recovery and EA was performed for 7 days. In vivo recording was performed at the end of EA treatment. (**I**) Alpha band in local field location recording. EA modulated the abnormal elevations in the alpha, bands. Sham group: (0.93 ± 0.10)*10^–6^, Model group: (7.32 ± 0.88)*10^–6^, Model + EA group: (4.98 ± 0.59)*10^–6^. (Kruskal–wallis test, ** vs. the Model + EA group, *** Sham group vs. Model + EA group, **** vs. Sham group). (**J**) Delta band in local field location recording. EA modulated the abnormal elevations in the delta, bands. Sham group: (0.30 ± 0.03)*10^–4^, Model group: (4.78 ± 1.01)*10^–4^, Model + EA group: (0.40 ± 0.06)*10^–4^. (Kruskal–wallis test, **** vs. Sham group and Model + EA group, respectively). (**K**) Theta band in local field location recording. EA modulated the abnormal elevations in the theta bands. Sham group: (0.29 ± 0.03)*10^–5^, Model group: (2.52 ± 0.33)*10^–5^, Model + EA group: (1.45 ± 0.17)*10^–5^. (Kruskal–wallis test, ** Sham group vs. Model + EA group, **** vs. Sham group and Model + EA group, respectively). (I-K) *N =* 3 for Sham, Model, Model + EA group. Data are presented as mean ± SEM

The endocannabinoid system was suggested to be inseparable from the pathogenesis of pain and involved in the regulation of neuronal activity [35–37]. In this case, we tested whether endocannabinoid signalling in the ACC was associated with the EA-mediated analgesic effect. We first measured the expression of mRNA encoding CB1R in the ACC by quantitative real-time PCR (q-PCR). The results showed that the mRNA level was significantly downregulated, while EA markedly enhanced the expression (Fig. 2A). We further analysed the expression of CB1R protein by western blot (WB) and observed that EA obviously reversed the decrease in CB1R protein levels in the pain mice (Fig. 2B-C). Accordingly, the fluorescence intensity, area, and volume of CB1R were lower, and EA showed a significant enhancement (Fig. 2D-G). Taken together, these results indicated that CB1R in the ACC could contribute to EA-mediated analgesia. For preclinical and clinical studies that have shown that endocannabinoids are associated with the theta bands of neural oscillations [38–40], we further recorded local field potentials (LFPs) using in vivo electrophysiological recording (Fig. 2H). The pain model mice showed a significant enhancement in the alpha (9–13 Hz), delta (0–3 Hz) and theta (4–8 Hz) bands compared to those in the sham group, while EA modulated the abnormal elevations (Fig. 2I-K). Therefore, we speculated that the endogenous cannabinoid system in the ACC was involved in EA analgesia.

**Fig. 3:**
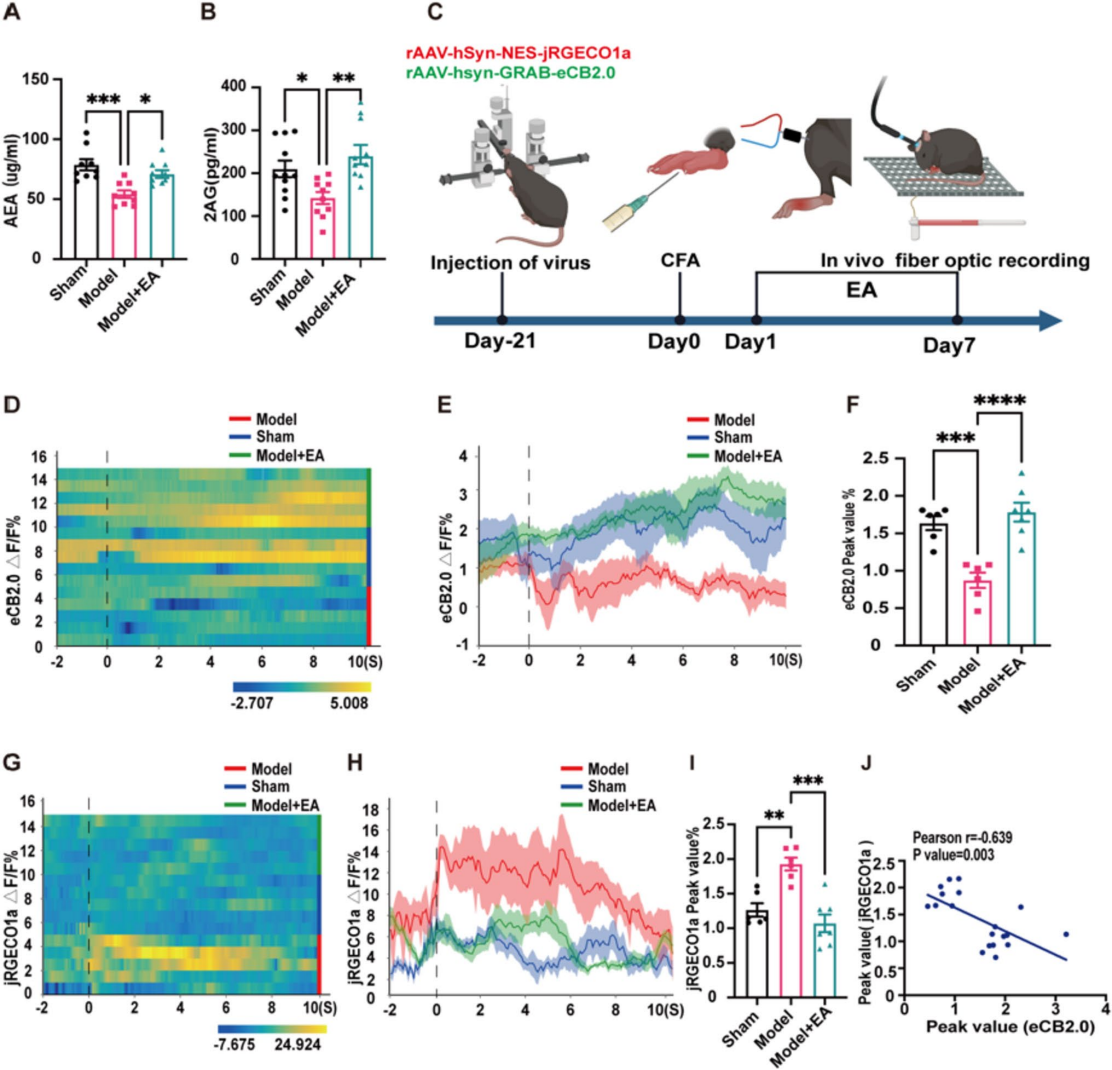
Electroacupuncture may reduce calcium excitability in the ACC via endogenous cannabinoids. (**A**) AEA content in ACC was determined by ELISA kit. EA significantly increased the expression level of AEA in the ACC of model mice. Sham group: 78.68 ± 4.57, Model group: 54.13 ± 3.17, Model + EA group: 70.89 ± 3.26. (One-way ANOVA with Bonferroni test, F _(2,24)_ = 11.36, *P* < *0.001.* *N =* 9 mice for each group. * vs. Model + EA group, *** vs. Sham group). (**B**) 2-AG content in ACC was determined by ELISA kit. EA significantly increased the expression level of 2AG in the ACC of model mice. Sham group: 209.98 ± 19.37, Model group: 142.18 ± 13.98, Model + EA group: 239.51 ± 20.81. (One-way ANOVA with Bonferroni test, F _(2,28)_ = 7.148, *P* < *0.01.* *N =* 11, 10, 10 for Sham, Model, Model + EA group. * vs. Sham group, ** vs. Model + EA group). (**C**) A timeline of in vivo multichannel fiber optic recording. We injected rAAV-hSyn-NES-jRGECO1a and rAAV-hsyn-GRAB-eCB2.0-WPRE viruses in the ACC and implanted fiber optics on day-21. After CFA injection on day 0, 7 days of EA was performed, and optical fiber recording was performed after the end of EA. (**D**) Heat maps showed changes in eCB signal activation between the three groups before and after 1.4 g von Frey stimulation in mice. (**E**) Line plots show changes in eCB signal activation between the three groups before and after stimulation. (**F**) Statistical plots showed the peak value of eCB signals in response to induced stimulation in mice after 7 days of EA. EA increases the level of eCB signals response in the ACC of model mice. Sham group: 1.64 ± 0.09, Model group: 0.87 ± 0.1, Model + EA group: 1.78 ± 0.13. (One-way ANOVA with Bonferroni test, F _(2,16)_ = 18.84, *P* < *0.001.* *N =* 6, 6, 7 for Sham, Model, Model + EA group. *** vs. Sham group, **** vs. Model + EA group). (**G**) Heat maps showed changes in jRGECO1a signal activation between the three groups before and after 1.4 g von Frey stimulation in mice. (**H**) Line plots show changes in jRGECO1a signal activation between the three groups before and after stimulation. (**I**) Statistical plots showed the peak value of jRGECO1a signals in response to induced stimulation in mice after 7 days of EA. EA reduces the level of jRGECO1a signals response in the ACC of model mice. Sham group: 1.27 ± 0.97, Model group: 1.93 ± 0.92, Model + EA group: 1.07 ± 0.13. (Kruskal–wallis test, *N =* 6, 6, 7 for Sham, Model, Model + EA group. ** vs. Sham group, *** vs. Model + EA group). (**J**) Linear correlation analysis of the correlation between eCB signal and jRGECO1a signal. There was a strong negative correlation between eCB and Ca^2+^ signals, Ca^2+^ signals decreased with the increase of eCB and conversely when eCB decreased Ca^2+^ increased. (Pearson Correlation Analysis, *N =* 6, 6, 7 for Sham, Model, Model + EA group. Pearson r = -0.639, *P* < *0.01*). Data are presented as mean ± SEM

Then, to directly explore the relationship of endocannabinoid signalling and neuronal hyperactivity in the ACC, we first performed enzyme-linked immunosorbent assay (ELISA) to determine the two main ligands of endogenous cannabinoids, anandamide (AEA) and 2-arachidonoylglycerol (2-AG) [24]. The levels of both ligands in the pain mice showed a significant decrease compared to that in the Sham group, while the levels of ligands in the EA group increased remarkably (Fig. 3A-B and Fig. S3 A-D). Then, to determine whether this impaired endocannabinoid signalling was associated with hyperactivity in the ACC, we recorded the functional changes in endogenous cannabinoids and Ca^2+^ signals simultaneously by in vivo multichannel optical fibre recording (Fig. 3C and Fig. S2 F). We observed a decrease in the endocannabinoid (eCB) and a significant increase in the Ca^2+^ signal in the ACC, while EA rescued this pathology (Fig. 3C-I). A correlation analysis of the recorded eCB and Ca^2+^ signals was performed, and the results showed that there was a strong negative correlation between eCB and Ca^2+^ signals (Fig. 3J). The above results suggested that impaired endocannabinoid signalling might contribute to the hyperactivity of pyramidal neurons in the ACC.

### The Role of CB1R in ACC was Necessary in the EA-mediated Analgesic Effect

**Fig. 4:**
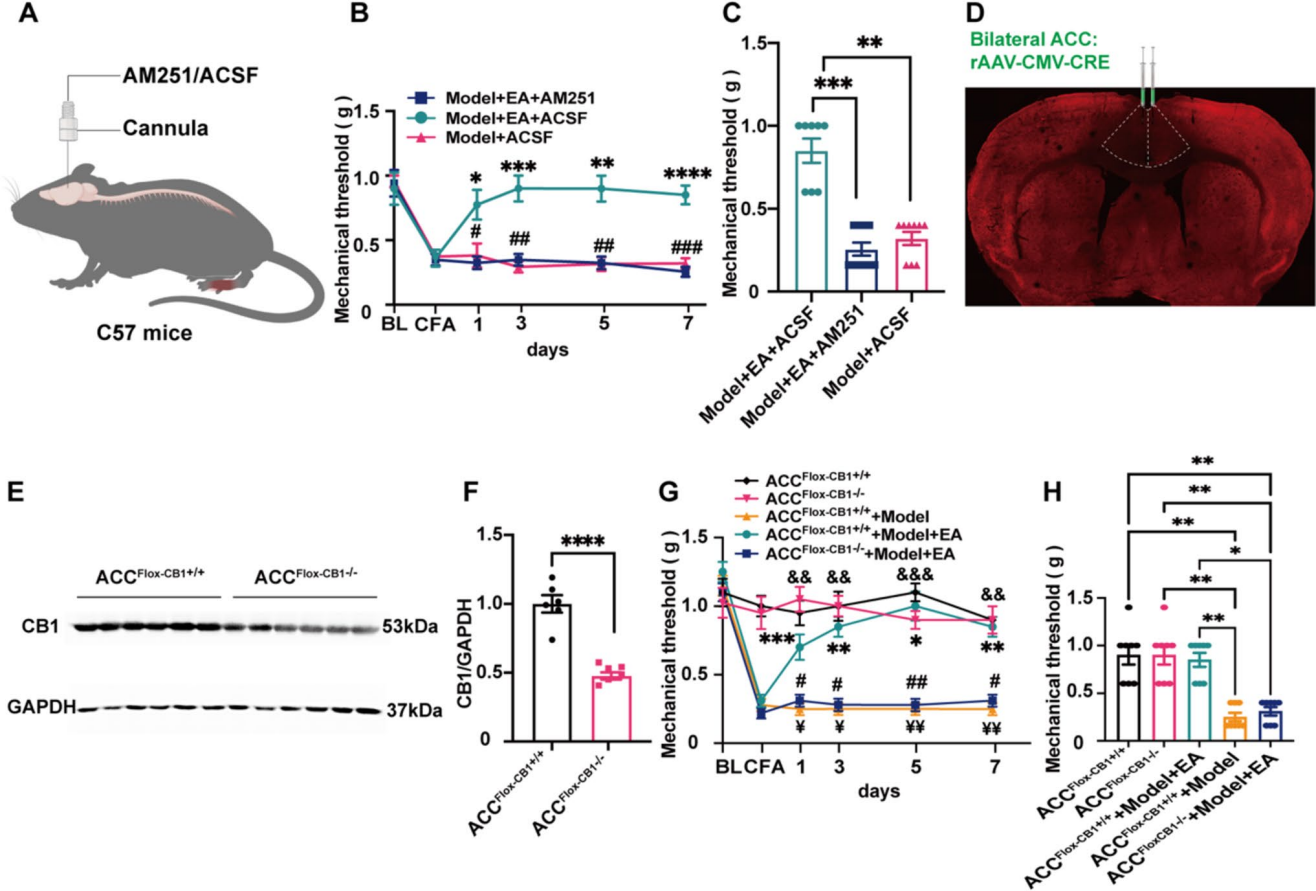
CB1R in ACC was necessary in the analgesic effect of electroacupuncture. (A) Schematic injection of AM251 or ACSF (300 μl) into ACC through cannula. (**B**) The mechanical threshold after EA was determined after injection of AM251 into ACC. Injection of AM251 in ACC reverses EA-mediated analgesia. (Two-way ANOVA with Bonferroni test, F _(10, 120)_ = 6.04, *P* < *0.0001.* *N =* 8, 10, 9 for Model + EA + ACSF, Model + EA + AM251 and Model + ACSF group. *, **, ***, **** vs. Model + EA + AM251 group. #, ##, ### vs. Model + ACSF group). (**C**) Statistical graph showing mechanical threshold determination on day 7 of EA after AM251 injection into ACC. Injection of AM251 in ACC reversed the effects of EA-mediated analgesia at day 7. Model + EA + ACSF: 0.85 ± 0.07, Model + EA + AM251: 0.26 ± 0.04 and Model + ACSF group: 0.32 ± 0.04. (One-way ANOVA with Bonferroni test, F _(2, 24)_ = 39.2, *P* < *0.0001.* *N =* 8, 10, 9 for Model + EA + ACSF, Model + EA + AM251 and Model + ACSF group. **, *** vs. Model + ACSF and Model + EA + AM251 group, respectively). (**D**) Immunofluorescence images showed the CB1R expression after bilateral ACC injection with rAAV-CMV-CRE knockout CB1R. (**E**) Representative gel image of CB1R protein levels in ACC after knockout. (**F**) Statistical diagram of relative expression level of CB1R protein in ACC of knockout mice. ACC^Flox−CB1+/+^ group: 1.0 ± 0.06, ACC^Flox−CB1−/−^ group: 0.48 ± 0.03. (Independent-samples T test, t _(10)_ = 7.68. *N =* 6 mice for each group. **** vs. ACC^Flox−CB1+/+^ group). (**G**) Mechanical threshold was used to determine the change of EA for 7 days after knocking down CB1R in ACC. Knockout of CB1R in ACC reverses the effects of EA-mediated analgesia. (Two-way ANOVA with Bonferroni test, F _(20, 175)_ = 9.08, *P* < *0.0001.* *N =* 8 for each group. ¥, ¥¥ ACC^Flox−CB1+/+^  + Model vs. ACC^Flox−CB1+/+^  + Model + EA group. #, ## ACC^Flox−CB1−/−^ + Model + EA vs. ACC ^Flox−CB1+/+^  + Model + EA group. *, **, *** ACC ^Flox−CB1−/−^ vs. ACC ^Flox−CB1+/+^  + Model and ACC ^Flox−CB1−/−^ + Model + EA group, respectively. &&, &&& ACC ^Flox−CB1+/+^ group vs. ACC ^Flox−CB1+/+^  + Model and ACC ^Flox−CB1−/−^ + Model + EA group, respectively). (**H**) Mechanical threshold was determined in ACC-CB1R knockout mice on day 7 of EA. Knockout of CB1R in ACC reverses the effects of EA-mediated analgesia at day 7. ACC ^Flox−CB1−/−^ group: 0.9 ± 0.1, ACC ^Flox−CB1+/+^ group: 0.9 ± 0.1, ACC ^Flox−CB1+/+^ + Model + EA group: 0.85 ± 0.73, ACC ^Flox−CB1−/−^ + Model + EA group: 0.31 ± 0.44, ACC ^Flox−CB1+/+^  + Model group: 0.25 ± 0.44. (One-way ANOVA with Bonferroni test, F _(4, 35)_ = 18.84, *P* < *0.0001.* *N =* 8 for each group. *, ** ACC ^Flox−CB1−/−^ + Model + EA group vs. ACC ^Flox−CB1+/+^, ACC ^Flox−CB1−/−^ and ACC ^Flox−CB1+/+^  + Model + EA group, respectively. ACC ^Flox−CB1+/+^  + Model group vs. ACC ^Flox−CB1+/+^, ACC ^Flox−CB1−/−^, and ACC ^Flox−CB1+/+^  + Model + EA group, respectively). Data are presented as mean ± SEM

Next, we validated the role of endocannabinoid signalling in the EA-mediated analgesic effect with pharmacological and genetic manipulations. We locally injected the CB1R antagonist AM251 (4.50 mM, 300 nl) [29] (Fig. 4A). The effect of EA was blocked by the application of AM251 (Fig. 4B-C). Moreover, we used ACC-CB1 KO mice to specifically downregulate CB1R in ACC [27]. The expression of CB1R was significantly reduced in the ACC ^Flox−CB1−/−^ group (Fig. 4D-F). There was no difference in the mechanical pain threshold between the ACC ^Flox−cb1−/−^ group and the ACC ^Flox−cb1+/+^ group suggesting that knockout of CB1R in the ACC does not affect baseline thresholds. However, the mechanical pain threshold was significantly reduced in the ACC ^Flox−cb1+/+^  + Model group, while the pain sensitization was reversed in the ACC ^Flox−cb1+/+^  + Model + EA group. However, the therapeutic effect of the ACC ^Flox−cb1−/−^ + Model + EA group disappeared (Fig. 4G-H). These results indicated that CB1R in the ACC was necessary for EA-mediated analgesia.

### The CB1R-mediated Downregulation of NR1 Expression was Associated with the EA-mediated Analgesia

**Fig. 5:**
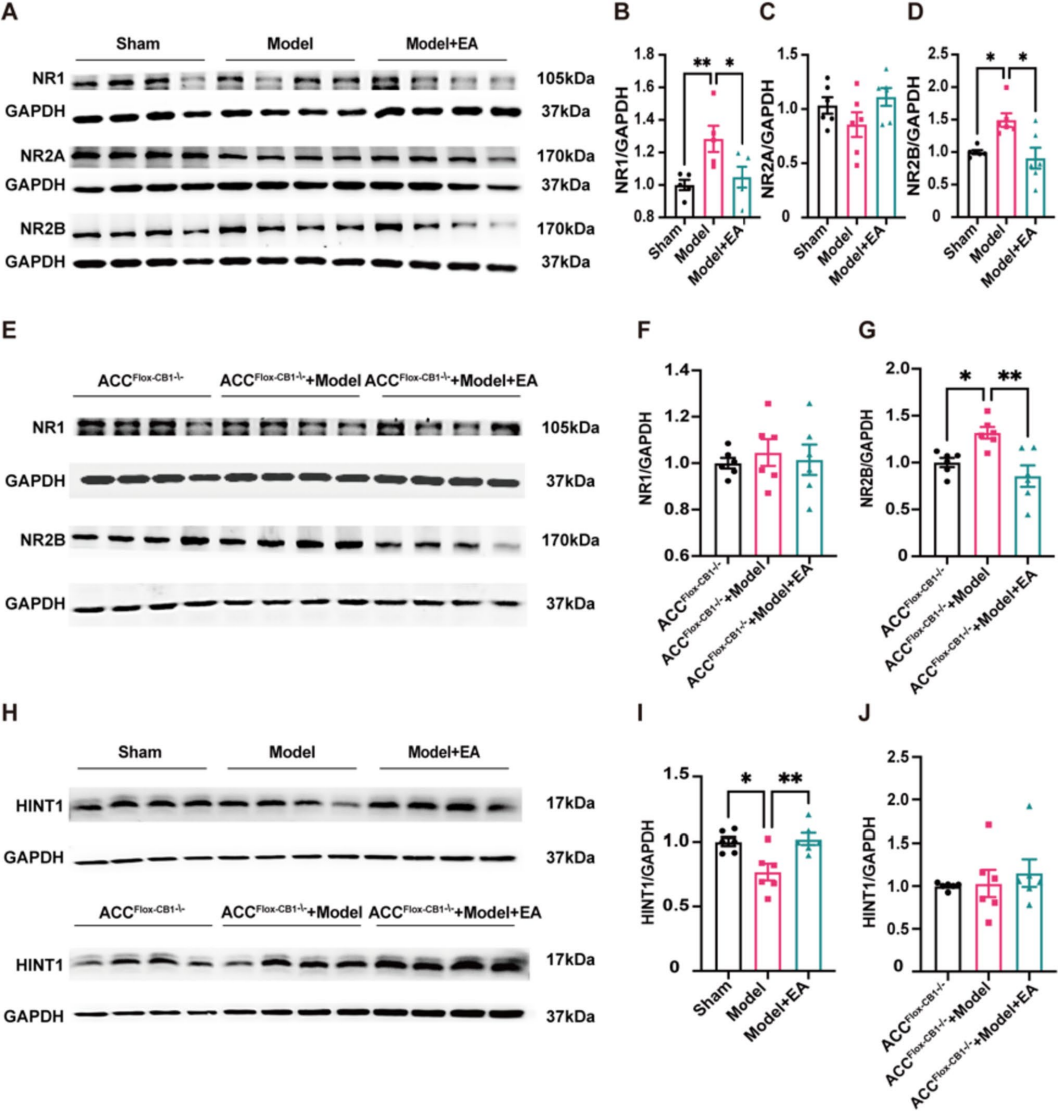
Electroacupuncture inhibits NMDAR through the interaction between CB1R and NR1 in ACC. (**A**) Representative gel image of NR1, NR2A, and NR2B protein levels in ACC. (**B**) Statistical diagram of relative expression level of NR1 protein in ACC of mice. EA reduces elevated NR1 protein expression in the ACC of model mice. Sham group: 1.0 ± 0.03, Model group: 1.28 ± 0.08, Model + EA group: 1.05 ± 0.06. (One-way ANOVA with LSD test, F _(2,12)_ = 5.95, *P < *0.05*. *N =* 5 for each group. * vs. Model + EA group, ** vs. Sham group). (**C**) Statistical diagram of relative expression level of NR2A protein in ACC of mice. EA did not produce differences in NR2A expression in the ACC of the three groups of mice. Sham group: 1.03 ± 0.08, Model group: 0.86 ± 0.11, Model + EA group: 1.11 ± 0.08. (One-way ANOVA with Bonferroni test, F _(2,15)_ = 1.99, *P* > *0.05.* *N =* 6 for each group. (**D**) Statistical diagram of relative expression level of NR2B protein in ACC of mice. EA reduces elevated NR2B protein expression in the ACC of model mice. Sham group: 1.0 ± 0.03, Model group: 1.49 ± 0.10, Model + EA group: 0.91 ± 0.16. (Tamhane T2 test, *N =* 6 for each group. * vs. Sham group and Model + EA group). (**E**) Representative gel image of NR1, and NR2B protein levels in mice with knockout of CB1R in ACC. (**F**) Statistical diagram of relative expression level of NR1 protein in mice with knockout of CB1R in ACC. Disappearance of EA effects on NR1 protein in three groups of mice after knockout of CB1R in ACC. ACC^Flox−CB1−/−^ group: 1.0 ± 0.02, ACC^Flox−CB1−/−^ + Model group: 1.04 ± 0.06, ACC^Flox−CB1−/−^ + Model + EA group: 1.01 ± 0.07. (One-way ANOVA with Bonferroni test, F _(2,15)_ = 0.202, *P* > *0.05.* *N =* 6 for each group.). (**G**) Statistical diagram of relative expression level of NR2B protein in mice with knockout of CB1R in ACC. EA still reduces elevated NR2B in model mice after knockout of CB1R in ACC. ACC^Flox−CB1−/−^ group: 1.0 ± 0.05, ACC^Flox−CB1−/−^ + Model group: 1.32 ± 0.06, ACC^Flox−CB1−/−^ + Model + EA group: 0.86 ± 0.11. (One-way ANOVA with Bonferroni test, F _(2,15)_ = 8.77, *P* < *0.01.* *N =* 6 for each group. * vs. ACC^Flox−CB1−/−^ group, ** vs. ACC^Flox−CB1−/−^ + Model + EA group). (**H**) Representative gel images of HINT1 protein levels in ACC in WT mice and mice that have knocked out the CB1R in ACC. (**I**) Statistical diagram of relative expression level of HINT1 protein in ACC of mice. EA was able to increase the decrease of HINT1 in the ACC of model mice. Sham group: 1.0 ± 0.03, Model group: 0.77 ± 0.07, Model + EA group: 1.02 ± 0.04. (One-way ANOVA with Bonferroni test, F _(2,15)_ = 8.169, *P* < *0.01.* *N =* 6 for each group. * vs. Sham group, ** vs. Model + EA group). (**J**) Statistical diagram of relative expression level of HINT1 protein in mice with knockout of CB1R in ACC. Disappearance of EA effects on HINT1 protein in three groups of mice after knockout of CB1R in ACC. ACC^Flox−CB1−/−^ group: 1.0 ± 0.02, ACC^Flox−CB1−/−^ + Model group: 1.03 ± 0.16, ACC^Flox−CB1−/−^ + Model + EA group: 1.15 ± 0.16. (One-way ANOVA with Bonferroni test, F _(2,15)_ = 0.374, *P* > *0.05.* *N =* 6 for each group). Data are presented as mean ± SEM

Activation of CB1 signalling could weaken the expression/function of NMDARs through presynaptic and postsynaptic pathways [41, 42]. As one of the major ionotropic glutamate receptors, NMDARs in the ACC play an important role in injury-induced changes in synaptic plasticity [43, 44]. Thus, we first examined the expression of NR1, NR2A and NR2B. EA downregulated the pain-induced increase in NR1 and NR2B, while the expression of NR2A was comparable among these groups (Fig. 5A-D). Next, we tested whether this alteration was associated with endocannabinoid signalling. The expression of NR1 and NR2B subunits in the ACC-CB1 KO mice with rAAV-CMV-CRE virus injection into the ACC was examined. The expression of NR2B was still elevated in the model mice, and this trend was reversed after EA, while there was no significant difference in the expression of NR1 among the three groups (Fig. 5E–G). These results suggest that NR1 may be involved in the endogenous cannabinoid signalling pathway regulating EA-mediated analgesia.

Several studies have suggested that cannabinoids inhibit NMDAR activity by binding CB1R to the NR1 subunit via the HINT1 protein, which promotes CB1R cointernalization with the NR1 subunit [45]. Therefore, we then measured the HINT1 protein in the ACC. The results revealed that the expression of HINT1 in the model mice showed a trend of downregulation, while EA increased the expression of HINT1 (Fig. 5 H, I). We examined the expression of HINT1 in the ACC of ACC-CB1 KO mice and found no difference in HINT1 expression among the three groups (Fig. 5 H, J). The above results suggested that CB1R and HINT1 may be jointly involved in the process of EA analgesia.

## Materials and Methods

### Animals

Male C57BL/6 J (8–10 weeks, 20–25 g) mice were obtained from the Laboratory Animal Center of Guangzhou University of Traditional Chinese Medicine. Mice carrying the ‘floxed’ CB1R–expressing gene (Cnr1f/f) were generously donated by Prof. Man Li [28]. The GAD67-GFP knock-in mice were from Professor Yongjun Chen. All mice (5 per cage) were housed in a standard laboratory room with temperature of 23–25 °C and humidity of 50%-60% and kept on a 12-h daily light–dark cycle (lights on from 7:00 to 19:00). Water and food were available ad libitum.

### Establishment of the Pain Model

We examined the baseline mechanical threshold and latency of the mice by von Frey and infrared thermal radiation before modelling, and mice with a baseline outside the normal range were excluded. Model mice developed inflammatory pain by injecting 25 µl of CFA (Sigma-Aldrich, St. Louis, MO) into the left plantar under 1.5% isoflurane anaesthesia, while sham mice were injected with the same dose of saline.

### Electroacupuncture Treatment

Anaesthetized mice were induced with 1.5% isoflurane and placed in a fixed mask. Two acupuncture needles (0.25 mm*13 mm) were inserted 2–3 mm into the left hindlimb ST36 and the interval between the two needles was 1 mm. The ST36 is in the area approximately 4 mm below the knee joint and approximately 2 mm lateral to the anterior tibial tuberosity [14]. The EA current was set at 1 mA and frequency of 2 Hz for 20 min for continuous 7 days by using HANS stimulator (HANS-200A/100B, Beijing, China) [46]. The Sham group was treated with isoflurane anaesthesia for the same time. For Sham EA, the acupuncture needles were fixed on the ST36 with adhesive tape without penetrating the skin and connecting electrodes [28].

### Von Frey Filament Test

The mechanical withdrawal threshold was assessed by von Frey fibres (Ugo Basile, Italy) as described previously [31]. The mice were acclimated to the environment for 30 min. After the mice were quiet, the fibre was aligned with the centre of the left plantar, the force was evenly applied to keep the fibre in a "c" shape for 5 s, and the mice were considered positive if they withdrew their feet. The test was performed sequentially using 0.16 g, 0.4 g, 0.6 g, 1.0 g, 1.4 g, and 2.0 g of fibre, and each gram was repeated 5 times with an interval of 1 min. If the same gram was positive 3 times, the gram was the mechanical threshold of the mice, and if it was negative 3 times, the next gram of fibre was replaced for the test. We tested mechanical thresholds in mice using von Frey on days -1, 0, 1, 3, 5, 7, 10 and 14.

### Thermal Latency Test

Hargreaves’s apparatus (Ugo Basile, Italy) was used to measure the thermal latency [47]. Before the test, the mice were adapted for 30 min, and then the infrared emitter was aimed at the left plantar centre of the mice. After starting the test, the apparatus will perform automatic timing until the mice withdraw their paws and then will stop timing. Each mouse was measured 3 times to take the average value, with each interval of 5 min.

### Open Field Test

As described previously [48], before the formal test, the mice were placed in the laboratory 30 min in advance to acclimatize to the environment, and then placed individually in the open field of 50 * 50 * 50 cm located in the soundproof box for 10 min. A video camera was used to record the movement trajectory, and the total distance and centre time of the mice were analysed by software at the end.

### Virus Injection

Mice were anaesthetized by intraperitoneal injection of 1.25% Avertin (20 ml/kg). The scalp was cut longitudinally approximately 1 cm to expose the skull and the periosteum was cleaned. The mice were then attached to the adaptor and the ACC (AP: + 0.8 mm; ML: -0.25 mm; DV: -1.0 mm) was located by a stereotaxic instrument (RWD, China). After injection (300 nl, 30 nl/min), the needle was kept in place for 10 min.

To perform in vivo two-photon calcium imaging, we injected rAAV-CaMKIIα-GCaMP6fs-WPRE virus and performed imaging after 3 weeks for recovery. For in *vivo* multichannel optical fibre recording, 300 nl rAAV-hSyn-NES-jRGECO1a and rAAV-hsyn-GRAB_eCB2.0-WPRE were injected simultaneously to label Ca^2+^ and endogenous cannabinoid signals, respectively. To achieve specific knockout of CB1R expression in the ACC, we injected rAAV-CMV-CRE into the bilateral ACC of mCnr1flox mice. To achieve dual immunofluorescence staining of CaMKIIα neurons and CB1R, we labelled CaMKIIα neurons by injecting rAAV-CaMKIIα-EGFP-WPRE. All viruses were sourced from Pivotal Technology Wuhan Pivotal Brain Science and Technology Co., Ltd.

### *In vivo* Two-photon Calcium Imaging

After virus injection on the stereotaxic instrument, the area around the injection site was disinfected with iodophor, followed by the application of a light-curing adhesive on the surface of the skull and the use of fluid resin (3 M, American) to fix a clear glass ring on the skull, and the mice were moulded and treated with EA after 21 days of recovery. The mice were recorded using a two-photon microscope. The software that comes with the Nikon two-photon microscopy imaging system (AIR MP, Nikon) was used for data collection (910 nm, 25X). Fluorescence changes in the spontaneous network activity of neurons in the ACC were observed using sapphire (Chameleon Vision II) laser excitation of CaMKIIα neurons expressing GCaMP6fs in awake mice. The laser power under the objective lens is 10–28 mW. A microscope equipped with a 25 × NA 1.05 objective lens (Nikon) was used for imaging at 512 * 512 pixels (0.994 µm/pixel), and a microscopic imaging system (NIS-Elements AR 4.60, Nikon) was used for calcium activity acquisition with a frame rate of 3.9 Hz. Recordings were performed 10 min in the 7 days after modelling, and the recordings were performed at the same time point in each group.

### *In vivo* Electrophysiological Recording

The mice were anaesthetized with 1.5% isoflurane and fixed on the adapter. The electrodes were then implanted vertically at a depth of 1 mm at a speed of 5 μm/s into the ACC. Then model-making and EA treatment was performed, and electrophysiological data were acquired by recording for 10 min using an electrophysiological recording system (MEDUSA, BIO-singnatechnologies) with a sampling frequency of 4000 Hz and a filter of 200 Hz. Electrophysiological data were analysed using Neuroexplorer (Plexon, USA). The power density of the broadband signal was calculated, for the full frequency band (0 ~ 100 Hz). The average power spectral density was calculated, using the data recorded before modelling as a baseline.

### *In vivo* Multichannel Optical Fibre Recording

After virus injection, the optical fibre core (Braintech, China) was implanted in the right ACC of mice through stereotaxic surgery, to record Ca^2+^ signals and eCB signals. The jumper was connected to the multichannel optical fibre recording system (RWD, China), and the mice were placed in a cage on wire rack to acclimate for 30 min. Fluorescence signals jRGECO1a and eCB2.0 were activated by excitation beams of 560 nm LED and 470 nm LED, respectively. To induce a pain response in CFA mice during the recording period, 1.4 g von Frey was used to stimulate the left plantar of mice for 3 s and the corresponding time points were recorded. The stimulation was repeated 3 times, each with an interval of 1 min. By calculating (F − F0)/F0, we obtain the values of the change in the Ca^2+^ signal and eCB (ΔF/F), which are expressed as an average or heatmap. We used MATLAB 2021 software for calculation. In detail, ΔF is the average value of jRGECO1a and eCB2.0 signals within 10 s after stimulation minus 2 s before stimulation, while F represents the signal changes before and after stimulation.

### Immunohistochemistry and Imaging

Mice were injected intraperitoneally with 1.25% avertin (20 ml/kg) for anaesthesia. The mice were perfused with 0.9% saline and PFA and brain tissue was extracted and soaked in 4% PFA at 4 °C overnight, followed by gradient dehydration with 15% and 30% sucrose solutions. Tissue-Tek OCT (Sakura, Japan) was used to bury the brain tissue, and the slices were cut into slices with a thickness of 40 μm by a cryotome (Thermo, Germany). The slices were washed with PBS 3 times, soaked in blocking buffer (5% goat serum containing 0.3% Triton X-100) and incubated in a 37 °C water bath for 1 h. Anti-c-Fos (Guinea pigs, SYSY) and anti-CB1R (rabbit, Abcam) antibodies were diluted with blocking buffer solution at a ratio of 1:500. After the slices were washed in PBS 3 times, they were incubated with secondary antibodies including anti-rabbit Alexa 594 (1:500, Thermo Fisher Scientific), and anti-guinea pigs Alexa 488 (1:500, Abcam) at 37 °C for 2 h. In the last stage, the slices were mounted onto microslides and stained with 4,6-diamidino-2-phenylindole (DAPI:1 μg/5 ml, Sigma) for 10 min. A laser confocal microscope (Nikon A1 Confocal System, Japan) was used for fluorescence imaging of the slices.

### Western Blot

The ACC brain tissue was added to RIPA lysis buffer and protease inhibitor cocktail for homogenization and centrifugation, and the supernatant was collected. The concentration of the sample was measured using the BCA protein assay kit (Thermo, Germany) and quantified. The protein was denatured by adding SDS-PAGE Protein Loading Buffer to the sample and heating in a 95 °C water bath for 10 min. The protein samples were separated on an 8% glycine-SDS-PAGE gel, and electrophoresed at 70 v for 30 min, and then adjusted to 120 v for 60 min. The protein was transferred to PVDF membrane by the Trans-Blot Turbo system (Bio-Rad Laboratories, United States). After the membrane was washed with TBST solution, the membrane was blocked at room temperature for 15 min with Quick Blocking Buffer (Beyotime, China). After washing again, the membrane was soaked in primary antibodies, including anti-CB1R (1:1000, rabbit, Abcam), anti-NR2A (1:1000, rabbit, CST), anti-NR2B (1:1000, rabbit, CST), anti-NR1 (1:1000, rabbit, Abcam), and anti-HINT1(1:1000, rabbit, Abcam), and placed in a 4 °C refrigerator overnight. Finally, after the membrane was washed, the goat anti-rabbit HRP-conjugated secondary antibodies (1:10000, Abcam) were incubated at room temperature for 2 h. The membrane was immersed in enhanced chemiluminescence (ECL) luminescent solution (Meilunbio, China) and then western blotting was observed using a chemiluminescence system (Peiqing Technology Co, China).

### ELISA

The ACC brain tissue was homogenized in PBS and centrifuged, and the supernatant was extracted. The protein concentration of the sample was determined using the BCA Protein Assay Kit (Thermo, Germany). The contents of AEA and 2-AG in ACC and serum were detected by ELISA kit (MeiMian, China) and the operation was carried out in strict accordance with the instructions.

### Cannulation and Microinjection

Mice anaesthetized with 1.25% avertin (20 ml/kg) were fixed on a stereotaxic tube, the cannula (62,204, RWD, China) was embedded 1 mm deep in the right ACC brain region, and then the mice were placed in cages alone for 2 weeks to recover. Ten minutes before EA, 300 nl of CB1R antagonist AM251 was diluted to a concentration of 2.5 mg/mL (4.50 mM) using 100 μl Dimethyl sulfoxide (DMSO) + 400 μl PEG300 + 50 μl Tween-80 (MedChemExpress, United States) injected into the ACC region at a rate of 30 nl/min through a microinjection pump (RWD, China) connecting the cannula. The control group was injected with the same amount of artificial cerebrospinal fluid by the same method. The behavioural test was performed 30 min after EA.

### Quantitative Real-time PCR

ACCs were quickly removed after euthanasia, and TRIzol reagent was added to the samples for ultrasonic lysis. Chloroform was then added to the lysate for centrifugation to collect RNA precipitates and RNAse-free hydrolysate precipitates were added. The concentration and purity of RNA were determined by an ultramicro-UV spectrophotometer, and then the total RNA was reverse transcribed into cDNA by adding reaction mixture to the sample. The 7300 System was used for q-PCR, and the 7300 System Software was used to quantify the relative expression levels. The threshold cycle (CT) was used to determine the expression level of each gene. The amount of endogenous control (β-actin) normalized target mRNA was obtained by 2 CT scans. CB1rightward (GGGACTCAGACTGCCTGCACAAG), CB1forward (GCACGGTGACAGTCACTATTTTA), β-actin rightward (CGT TGA CAT CCG TAA AGA CC), β-actin forward (AAC AGT CCG CCT AGC AC).

### Statistical Analysis

The measurement data in the experimental data were expressed by mean ± SEM, and if the data satisfied normal distribution and variance chi-square, independent sample t-test was used for comparison between two groups; for comparison of three groups and above, one-way ANOVA test was used, and if variance chi-square was satisfied, LSD test or Bonferroni correction was used for two-way comparison. If variance chi-square was not satisfied, Tamhane test was used the nonparametric Kruskal–Wallis test was used for multiple group comparisons. All experimental data were statistically analysed by SPSS version 26.0, and *P* < *0.05* was considered a statistically significant difference between groups. Plotting was performed by GraphPad Prism 9.0.

## Discussion

Our study found that CB1R in the ACC played a crucial role in EA-mediated analgesia for inflammatory pain. First, EA can reduce mechanical and thermal allodynia and upregulate CB1R expression in the ACC. Second, EA inhibited the hyperactivity of pyramidal neurons, which could be associated with potentiation of endocannabinoid system. Third, the results from pharmacological and genetic manipulation of CB1R suggested the necessary role of CB1R in ACC for EA to exert the analgesic effect. Last, our results provide the evidence for EA-mediated modulation of CB1R by potentially downregulating NR1 via HINT1.

In clinical practice, chronic pain is complex and has a variety of different types and pathological bases, including chronic inflammatory pain and neuropathic [2]. In this study, the conclusion of hyperactivity in the ACC obtained from the pain model induced by CFA is consistent with previous reports from neuropathic pain models, such as chronic constriction injury of the sciatic nerve (CCI) and spared nerve injury (SNI), which involves abnormal excitation of pyramidal neurons in the ACC brain region [19, 20, 49]. Of course, this result needs to be verified in more types of pain models, such as cancer pain and visceral pain. In addition, endocannabinoids have been confirmed to participate in the analgesic process of S1, vlPAG, striatum and other brain regions in neuropathic pain and inflammatory pain [28, 32]; thus, our studies suggest that endocannabinoid signalling in the ACC is also involved in inflammatory pain, while its role in neuropathic pain needs to be further explored.

The ACC is involved in a wide range of cognitive and affective processing and is associated with affective pain such as anxiety, depression, and empathy [31, 50]. Several studies have shown that EA can reduce mechanical sensitization in different models through the ACC while modulating affective symptoms such as anxiety and depression [51–53]. However, in this study, the results obtained from the open field test demonstrated that the mice with CFA treatment after 7 d showed no difference in time in the centre among these groups, suggesting that these mice might represent no anxious or depressive-like phenotypes. The contradictory results may be attributed to the following reasons. First, the CFA-induced pain model takes more than 21 days to produce an anxiety-affective phenotype [54, 55], while the open field test was examined at 7 d for our study focusing on the analgesia [27, 28]. Second, a single open-field experiment may not be able to fully capture all the affective changes of animals. Although there is a long-standing controversy about how the ACC encodes and distinguishes the perception of pain and affective pain [56], some studies have proposed that the affective response of the ACC to pain is limited to the rostral part, while the perception of pain corresponds to the caudal part [17, 57]. Our results suggest that the ACC plays an important role in the perception of pain, which also supports the caudal region encoding the sensory components of pain. Of course, the affective part of pain is also very important and is the direction of our future exploration.

Studies have demonstrated that CB1R is abundantly expressed within the ACC and is distributed in both GABAergic and glutamatergic synapses [25]. To further investigate which neuronal type in the ACC is involved in EA-mediated analgesia, we performed immunofluorescence staining with CB1R colabelling in mice injected with rAAV-CaMKIIα-EGFP-WPRE in the ACC or GAD67-GFP transgenic mice. We found that EA increased the reduction in the colabelled area of CB1R and CaMKIIα neurons in the ACC induced by chronic inflammatory pain but decreased the enhancement of the colabelled area of CB1R and GAD67 neurons (Fig. S3 A-H). This result suggested that both excitatory and inhibitory neurons participated in the EA-mediated analgesic effect. In addition, excitatory neurons account for approximately 80% of neurons in L2/3 and L5 of the ACC [19], thus we primarily targeted excitatory neurons in the ACC during the in vivo two-photon imaging and optical fibre recording experiments. Notably, the role of inhibitory neurons, especially specific inhibitory neuronal types, including CCK interneurons, somatostatin (SST) and vasoactive intestinal peptide (VIP) interneurons in the ACC should be further investigated [25, 58]. A study demonstrated that EA could modulate Ca^2+^ activity of neural circuits in the S1 cortex dependent on CB1R activation, including inhibition of excitatory neurons, enhancement of SST interneurons, and inhibition of VIP interneurons [32]. However, whether there are similar neural circuits in the ACC that regulate neuronal hyperexcitability via CB1Rs on GABAergic interneurons deserves further study in the future.

Most of the current studies confirm that CB1R activation reduces glutamate release into the cleft and reduces overactivation of NMDAR [59]. In our study, the effect of EA on the downregulation of NR1 was lost after knocking out CB1R, while the effect on the downregulation of NR2B was not affected. Studies have confirmed that CB1R coimmunoprecipitates with the NR1 subunit, but not with the NR2/3 subunit, or only a small amount [45]. In addition, CB1R has been shown to negatively control NMDAR activity by coupling the HINT1 protein to the NR1 subunit [42, 60], which is similar to our results indicating that the regulatory effects of EA on NR1 and HINT1 proteins in CFA-induced pain mice are abolished after knockout of CB1R in ACC. Therefore, we suggest that EA may rescue hyperactivity in the ACC via CB1R regulating NR1 through HINT1.

Our study found an important role of CB1R in the ACC in EA-mediated analgesia, which may be induced by CB1R downregulating NR1 via HINT1. However, inhibitory neurons are important for controlling and balancing neuronal excitation in the central nervous system, and there are many types of inhibitory neurons; thus, investigating whether inhibitory neurons in the ACC are also involved in the endocannabinoid pathway for EA-mediated analgesia and the role of specific inhibitory neurons, such as somatostatin (SST) and vasoactive intestinal peptide (VIP) interneurons, and how they interact with excitatory neurons to restore cortical excitability is needed. In addition, more evidence to provide the direct structural/functional interaction among NR1, CB1R and HINT1 is also worthy to be exploring.

### Supplementary Information

Below is the link to the electronic supplementary material.

Supplementary file1 (DOCX 4931 KB)

## Data Availability

The original data in this study are presented in the text of the article or in the Supplementary Material; further inquiries can be made by contacting Lulu Yao and Nenggui Xu.

## Abbreviations

2-AG: 2-Arachidonoylglycerol
ACC: Anterior cingulate cortex
ACSF: Artificial cerebrospinal fluid
AEA: Anandamide
CB1R: Cannabinoid type 1 receptors
CCI: Chronic constriction injury of the sciatic nerve
CFA: Complete Freund's adjuvant
CNS: Central nervous system
DMSO: Dimethyl sulfoxide
EA: Electroacupuncture
eCB: Endocannabinoid
ECL: Enhanced chemiluminescence
Elisa: Enzyme-linked immune-sorbent assay
HINT1: Histidine triad nucleotide-binding protein 1
IASP: International Association for the Study of pain
IF: Immunofluorescence
LPBN: Lateral parabrachial nucleus
LFPs: Local field potentials
mPFC: Medial prefrontal cortex
NR1: NR1 subunits of NMDAR
PAG: Periaqueductal gray
PEA: N-palmitoylethanolamide
qPCR: Quantitative real-time PCR
RVM: Rostral ventromedial medulla
S1: Primary somatosensory cortex
SNI: Spared nerve injury
SST: Somatostatin
ST36: Zusanli acupoint
VIP: Vasoactive intestinal peptide
WB: Western blot
