# Supplementary material for: Electroacupuncture Exerts Analgesic Effects by Restoring Hyperactivity via Cannabinoid Type 1 Receptors in the Anterior Cingulate Cortex in Chronic Inflammatory Pain
Source: Mol Neurobiol. 2023 Nov 13;61(5):2949–63. doi: 10.1007/s12035-023-03760-7 (PMC11043129; doi:10.1007/s12035-023-03760-7)
Supplement: Supplementary file 1 — Supplementary file1 (DOCX 4931 KB) [file 12035_2023_3760_MOESM1_ESM.docx]

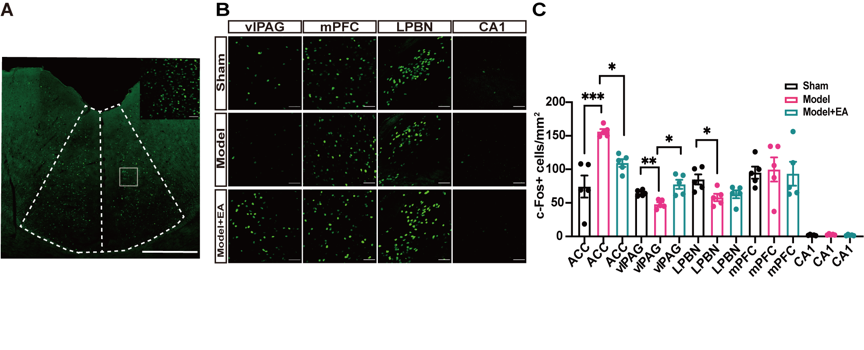


**S1 Electroacupuncture activates brain regions associated with CFA**

(A) Panorama and local magnification of Immunofluorescence staining image of c-Fos^+^ neurons in ACC region. Scale bars: 500 μm.

(B) Immunofluorescence staining images of c-Fos^+^ neurons in PAG, PFC, PBN and CA1 regions of three groups of mice. Scale bars: 50 μm.

(C) The Statistical diagram showed the number of c-Fos^+^ neurons in different brain regions of the three groups of mice. (One-way ANOVA with Bonferroni test, N = 5 for each group, *, **, *** vs. Sham group and Model + EA group). Data are presented as mean ± SEM.


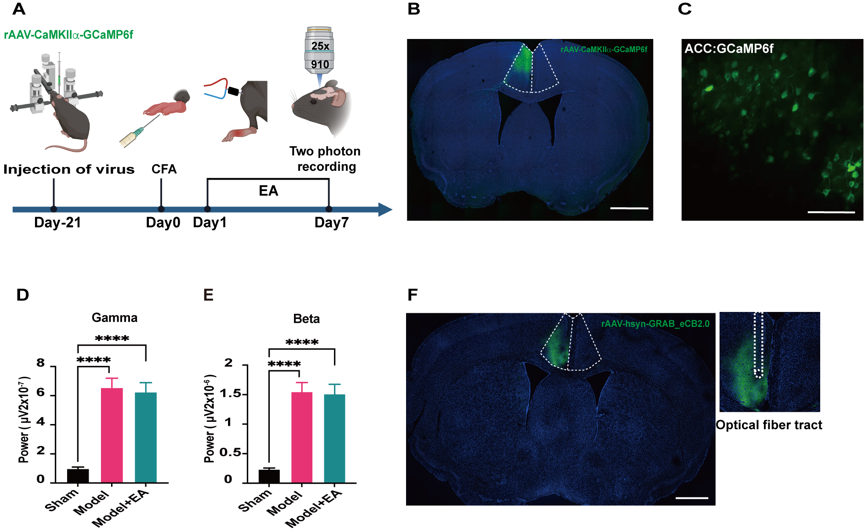


**S2 The changes of neuronal activity in ACC were recorded *in vivo***

(A) A timeline of *in vivo* two-photon Ca^2+^ imaging. AAV-CaMKIIα-GCaMP6fs virus was injected into the ACC on day -21, and CFA was injected into the plantar of the mice on day 0, followed by 7 days of EA, and *in vivo* two-photon Ca^2+^ imaging was performed at the end of EA treatment.

(B)The transfection site of AAV-CaMKIIα- GCaMP6fs virus in ACC. Scale bars: 1000 μm.

(C) Using a two-photon microscope to capture images of neurons expressing GCaMP6fs in ACC, Scale bars: 50 μm.

(D) Gamma band in local field location recording. Sham group: (0.96 ± 0.14)*10^-7^, Model group: (6.52 ± 0.67)*10^-7^, Model + EA group: (6.2 ± 0.68)*10^-7^. (Kruskal-wallis test, N = 3 mice for each group. **** vs. Model group and Model + EA group, respectively)

(E) Beta band in local field location recording. Sham group: (0.23 ± 0.03)*10^-6^, Model group: (1.54 ± 0.16)*10^-6^, Model + EA group: (1.50 ± 0.17)*10^-6^. (Kruskal-wallis test, N = 3 mice for each group. **** vs. Model group and Model + EA group, respectively)

(F) The transfection site of rAAV-hsyn-GRAB_eCB2.0 virus (left) and the optical fiber tract (right) in ACC. Scale bars: 1000 μm. Data are presented as mean ± SEM.


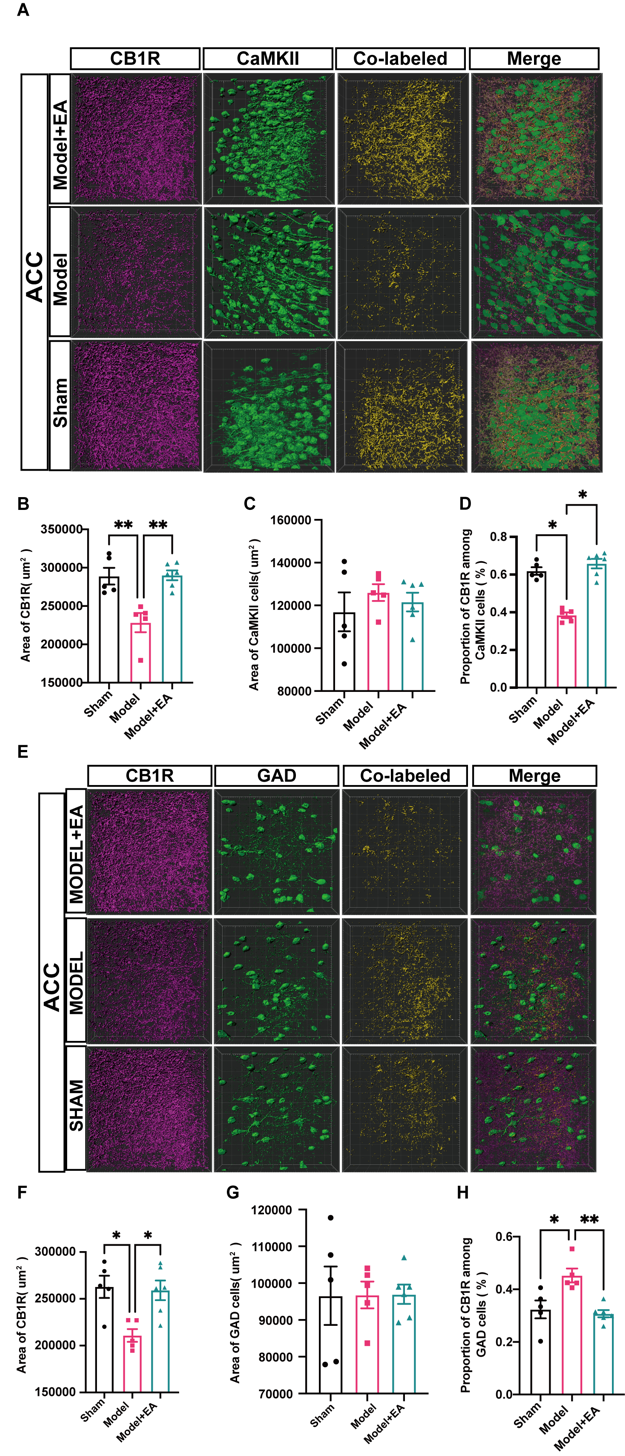


**S3 CB1R in ACC was co-labeled with** **CaMKIIα neurons and** **GAD67 neurons.**

(A) 3D-processed images of CB1R and CaMKIIα neurons co-labeled. Scale bars: 30 μm.

(B) The statistical plot represents the area of the CB1R in ACC, Sham group: 288972.2 ± 10964.12, Model group: 228390.2 ± 12538.06, Model + EA group: 290177.67 ± 6352.24. (One-way ANOVA with Bonferroni test, F _(2,13)_ = 12.40, *P <0.01.* N = 5, 5, 6 for Sham, Model and Model + EA group. ** vs. Sham group and Model + EA group, respectively).

(C) The statistical graph represents the area of the CaMKIIα neurons in ACC. Sham group: 116997 ± 9112.79, Model group: 126046.61 ± 3929.05, Model + EA group: 121609.24 ± 4346.65. (Tamhane T2 test, N = 5, 5, 6 for Sham, Model and Model + EA group).

(D) The statistical plots showed the proportion of the co-labeled area of CB1R and CaMKIIα neurons in the total CaMKIIα neurons (%). Sham group: 0.62 ± 0.02, Model group: 0.38 ± 0.15, Model + EA group: 0.66 ± 0.26. (One-way ANOVA with Bonferroni test, F _(2,13)_ = 6.05, *P <0.05.* N = 5, 5, 6 for Sham, Model and Model + EA group. * vs. Sham group and Model + EA group, respectively).

(E) 3D-processed images of CB1R and GAD 67 neurons co-labeled. Scale bars: 30 μm.

(F) The statistical plot represents the area of the CB1R in ACC, Sham group: 262789.01 ± 11894.76, Model group: 210871.1 ± 6846.25, Model + EA group: 259057.96 ± 10555.62. (One-way ANOVA with Bonferroni test, N= 5, 5, 6 for Sham, Model and Model + EA group. F _(2,13)_ = 7.86, * vs. Sham group and Model+ EA group, respectively).

(G) The statistical graph represents the area of the GAD 67 neurons in ACC. Sham group: 96575.81 ± 7939.87, Model group: 96792.72 ± 3631.82, Model + EA group: 96979.05 ± 2625.24 (Tamhane T2 test, N= 5, 5, 6 for Sham, Model and Model + EA group).

(H) The statistical plots showed the proportion of the co-labeled area of CB1R and GAD 67 neurons in the total GAD 67 neurons (%). Sham group: 0.32 ± 0.03, Model group: 0.45 ± 0.03, Model + EA group: 0.31 ± 0.01. (One-way ANOVA with Bonferroni test, F _(2,13)_ = 10.0, *P <0.01.* N = 5, 5, 6 for Sham, Model and Model + EA group. * vs. Sham group, ** vs. Model + EA group). Data are presented as mean ± SEM.
